# A New Digital Platform for Collecting Measurement Data from the Novel Imaging Sensors in Urology

**DOI:** 10.3390/s22041539

**Published:** 2022-02-16

**Authors:** Grzegorz Rybak, Krzysztof Strzecha, Marek Krakós

**Affiliations:** 1 Institute of Applied Informatics, Lodz University of Technology, ul. Stefanowskiego 18/22, 90-537 Łódź, Poland; strzecha@kis.p.lodz.pl; 2 Department of Pediatric Surgery and Urology, Hospital of J. Korczak in Łódź, 71 Piłsdskiego Av., 90-329 Łódź, Poland; marek.krakos@p.lodz.pl

**Keywords:** urology, tomography, UT, EIT, EHR, HL7, RIM, DICOM, microservices, IoT, message broker

## Abstract

The use of UT and EIT technologies gives the opportunity to develop new, effective, minimally invasive diagnostic methods for urology. The introduction of new diagnostic methods into medicine requires the development of new tools for collecting, processing and analysing the data obtained from them. Such system might be seen as a part of the electronic health record EHR system. The digital medical data management platform must provide the infrastructure that will make medical activity possible and effective in the presented scope. The solution presented in this article was implemented using the newest computer technologies to obtain advantages such as mobility, versatility, flexibility and scalability. The architecture of the developed platform, technological stack proposals, database structure and user interface are presented. In the course of this study, an analysis of known and available standards such as Hl7, RIM, DICOM, and tools for collecting medical data was performed, and the results obtained using them are also presented. The developed digital platform also falls into an innovative path of creating a network of sensors communicating with each other in the digital space, resulting in the implementation of the IoT (Internet of Things) vision. The issues of building software based on the architecture of microservices were discussed emphasizing the role of message brokers. The selected message brokers were also analysed in terms of available features and message transmission time.

## 1. Introduction

Functional disorders of the urinary tract are a common problem of the pediatric population. It is estimated that they may affect over 20% of 5-year-old children and 2–4% of adolescents. Urinary system defects in children are usually asymptomatic, therefore it is extremely important to regularly monitor the disease process and determine whether the patient can be treated conservatively or requires surgery.

Defects of the upper urinary tract, such as congenital hydronephrosis or obstruction of the uretero-bladder junction, undergoes spontaneous remission in about 80% of cases. One of them requires the systematic performance of tests to assess the secretory and excretory function of the kidneys [1]. The main test performed in this type of disease is dynamic renoscintigraphy. It is a safe test, but when it is performed, the isotope DMSA (dimercaptosuccinic acid) is administered intravenously. For this reason, a safe interval between follow-up examinations should always be considered. Often, it is not possible to maintain the intervals between examinations due to the unpredictable deterioration of the urinary tract, e.g., due to recurrent infections with fever, which leads to kidney damage.

In the case of other anatomical defects, such as duplication of the upper urinary tract, it is necessary to perform magnetic resonance imaging or computed tomography. Both of these diagnostic methods have significant limitations in young children. Magnetic resonance imaging must be performed under general anesthesia, while computed tomography is associated with the exposure of the child’s body to a very high dose of radiation [2].

The diseases of the lower urinary tract include both anatomical defects and functional disorders. Voiding cystouretrography (VCUG) is the “gold standard” for the anatomical evaluation of the bladder and urethra. Urodynamic examination is used to diagnose urinary tract dysfunctions. Both diagnostic methods are very invasive and often difficult to perform due to the child’s lack of cooperation during their performance. If it is necessary to repeat these tests, the child is exposed to doses of ionizing radiation (VCUG), the risk of urinary tract infections and damage to the lower urinary tract caused by the inserted catheter [3].

Diagnostic tests of the urinary system, while in adults are well accepted by the patient, in the case of children may be difficult to perform. Indications for repeating these tests to monitor the course of the disease must be carefully considered by a physician. Therefore, it is highly important to work on the development of new, less invasive diagnostic methods in order to obtain knowledge about the course of the child’s urinary tract disease.

The lack of non-invasive diagnostic methods and comprehensive analysis of the condition of the urinary tract reduces the likelihood of correct diagnosis and effective treatment. The results of the current measurement methods are unreliable. The use of the fusion of EIT (Impedance Tomography) and UT (Ultrasound Tomography) seems to be an ideal solution. The advantages of the prepared system include non-invasiveness, the possibility of long-term assessment of the condition of the urinary tract, and a relatively low price of the apparatus.

The aim of the project is to develop and implement into clinical practice non-invasive monitoring and diagnosis of urinary tract disorders in children using electrical impedance tomography (EIT) and ultrasound tomography (UT). At the beginning, the project required an analysis of medical processes, available standards for collecting medical data, preparation of the digital platform architecture, development of dedicated sensors and implementation of software development processes, including the preparation of the technological stack. The cases were covered and are extensively described in further chapters.

One of the non-invasive methods for cross-section monitoring is Electrical Impedance Tomography. This technique is based on various electrical properties for different types of materials. It has been proven that this kind of measurement is also relevant for biological tissue measurements. For this new imaging technique, a power or voltage is attached to the measured object in order to measure the current distribution or voltage distribution on the edge of the object. The complexity and advancement of this system has been emphasized in numerous studies, for example, indicating the use of FPGA systems in order to synchronize the detection and excitation of electrodes [4]. While data are gathered, the information is sent to a reconstruction algorithm, which allows the cross-section of the examined object to be computed. Although it seems easy, this technique has major difficulties, including low image resolution and nonlinear current flow [5].

**Figure 1:**
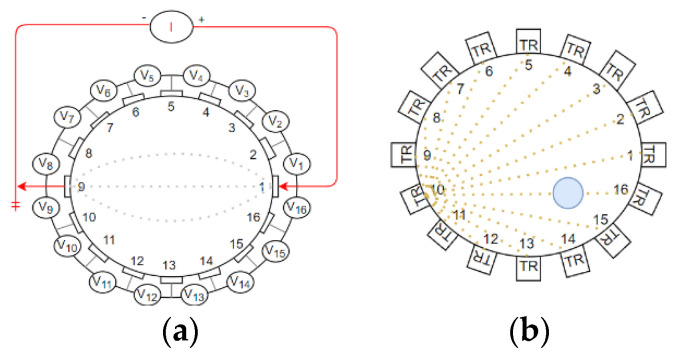
Tomography data collection models for selected sensors; (**a**) boundary potential for EIT with opposite sensors approach (**b**) Ultrasound Transmission Tomography.

Another technology which does not cause changes in the physical and chemical parameters that could interfere with the measurement results is UT (ultrasound tomography). Such a method also may be another non-invasive solution. Today, ultrasound is the main alternative to catheterization. With the use of simple methods, we are able to calculate bladder volume from scanned ultrasound images [6]. During measurement, only short waves are used and with their propagation and radiation properties, they can be treated as rays. The measurement of nonelectrical parameters allows, after relevant reconstruction transformations, the imaging of the internal structure of the studied medium, as well as flow parameters, which include velocity, average speed or velocity profile [5]. The models of tomography sensors are presented in Figure 1. The first image (a) shows EIT with an opposite sensor approach and measurement structure. The second one (b) presents several ultrasound transmitters placed around the examined body.

### Tomography Sensor Models Description

There are plenty of solutions allowing us to handle medical data. There are Orthanc, RadiAnt, SonicDICOM PACS, dcm4che, Dicoogle, EasyPACS, J-Pacs, OHIF, JVSdicom, PostDicom, MedDream PACS, HOROS Dicom, just to name the most important ones. Orthanc aims at providing a simple, yet powerful standalone DICOM server. It is designed to improve the DICOM flows in hospitals and to support research about the automated analysis of medical images. This solution is a well-known type of medical software, called the picture archiving and communication system—PACS.

The implementation of the PACS system is a prerequisite for keeping medical documentation only in digital form. As of 1 August 2014, this obligation is imposed on all entities providing health services by the Regulation of the Minister of Health of 21 December 2010, on the types and scope of medical documentation and the method of its processing (Poland, Journal of Laws of 2010, No. 252, item 1697) [7]. Unfortunately, not all computer platforms are generally free of charge. An important selection factor is the technology used to create the computer system. It is indicated that the best tools are those based on web technologies (websites, mobile applications), which can be transformed into so-called cloud solutions [8]. As it can be seen, the problem is significant and there is still a need for continuous development of the indicated tools.

In order to process medical data, the most common computer architecture is a layered architecture, which consists of the following blocks: measurement, communication, visualization, data persistence and inference, together with artificial intelligence (AI). From the point of view of communication between system modules, modern solutions are often based on Bluetooth and Internet technologies [9]. The use of this type of communication technology introduces many advantages, such as mobility, versatility, flexibility and scalability, which makes it a popular choice when choosing a technological stack of solutions. This allows a flexible choice of a distributed architecture, where the components are domain-oriented and communicate with each other using protocols.

Nowadays, we have plenty of decentralized architecture ideas. A relatively new concept, which extends the microservices and cloud computing approach, is the so-called fog computing. Fog computing puts a substantial amount of cloud computing facilities at the edge of a network, as opposed to establishing dedicated channels to a more centralized remote cloud infrastructure [10]. This kind of network node decomposition provides three major layers: things layer, fog layer and cloud layer. The devices used by the fog users are often considered resourceful in terms of their capabilities, but they are still incapable of executing certain complex tasks [11]. Fog computing facilitates these activities. Additionally, data sharing between computing nodes is crucial to deliver advanced artificial intelligence-based solutions. Often, the acquired sensor data are sent to the cloud computing layer, for instance data centers, in order to process so-called big data.

In cooperation with the industry and representatives of the medical sector, a system for collecting and automatically processing data for UT and EIT tomography is being prepared. The main contribution of this article is:

- Architectural proposition and implementation of a PAC system that reflects the IoT concept and enables automatic diagnosis based on the fusion of images of non-invasive medical measurements;
- The proposition of technical stack that provides critical functionality based on specialist requirements and architectural assumptions;
- Data model design for UT and EIT fusion images collection;
- Message brokers performance analysis.

## 2. The Concept of UT and EIT Measurement Platform

**Figure 2:**
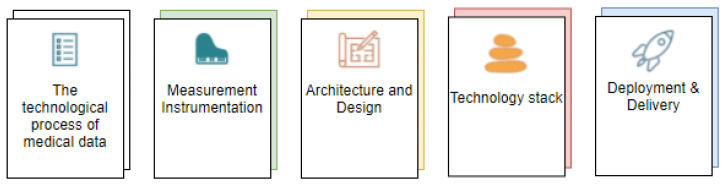
Selected aspects of creating a medical data collection system.

The creation of a digital platform for medical data collection is connected with many factors that directly affect the direction of the development process of such a system. The application is created in accordance with agile software development methodologies, where attention has been focused on the most important technological aspects. These contain technological processes of electronic health records (EHR), the measurement instrumentations, architecture for the novel digital platform, technology stack that must be established during development, as well as deployment and delivery cases. The main group of cases and development aspects are presented on Figure 2.

The main aspect from the list above is the process of collecting, storing and processing medical data. Depending on how the data is transferred and its characteristics, it is possible to outline the information flow path in the system while focusing attention on the process description. In the presented solution, there are two data transfer solutions: real time data acquisition and offline bulk load. This was feasible to achieve due to earlier prepared architecture, which is described below.

### 2.1. Architecture and Technical Stack

The architecture of microservices is a structural variant of service-oriented architecture (SOA), arranging the application as a set of loosely related services. The characteristic features of the architectural style of microservices are listed below:

− Services in microservice architecture (MSA) are recognized as processes that communicate using protocols such as HTTP [12,13];
− Services are organized around business values [14];
− Services can be implemented using a variety of programming languages, databases; hardware and software environment, regardless of technology [15].

The services are small, use messaging techniques, are limited by contexts, developed autonomously, independently implemented [15,16], decentralized, and made available through automated processes [16]. Due to the large number of services, decentralized continuous delivery and holistic monitoring are essential for the efficient development, maintenance and operation of such applications [17]. There are many benefits to decomposing an application into many smaller services, including:

− Modularity [15];
− Scalability [18];
− Integration of older systems [12,13,19] using an incremental approach [20];
− Distributed development through refactoring [21] and streamlining delivery processes [22].

The microservices approach also has disadvantages: services create information barriers, connections between services in the network have a higher cost in terms of transmission delay and message processing time than calls as part of a monolithic service process, testing and implementation are more complicated, transferring functionality between services is more difficult [16], the perception of service size as a structuring mechanism may lead to too many microservices, internal modularization may lead to a simpler design [23], and the commonly used protocol (HTTP) is not suitable for working with internal microservices, which often have to demonstrate a high level of reliability [24]. However, the aforementioned advantages largely outweigh the described disadvantages.

The most important technological choice is the way microservices communicate with each other (synchronous, asynchronous, UI integration) and the choice of protocols (RESTful HTTP, messaging, GraphQL, WebRTC). In a traditional system, most technology choices affect the entire system. Therefore, the approach to technology selection is different [25].

In the microservice architecture, all communication is routed through a group of brokers. These are programs that operate on the basis of advanced routing algorithms. Each microservice is connected to a broker. The service sending the information is the publisher and the recipient is the subscriber. The news is published in a specific topic channel. The subscriber receives such messages from the channels that he has subscribed to.

There are a number of brokers available today that provide a different spectrum of possibilities and functionalities. The messaging broker systems that meet the criteria of free license and high popularity are Apache Kafka, Mosquito, Hive, RabbitMQ, ActiveMQ, Apollo JMS and others.

The use of brokers also falls into an innovative path of creating a network of objects communicating with each other in the virtual space, resulting in the implementation of the IoT (Internet of Things) vision. Its idea is to ensure the possibility of exchanging messages between physical objects with or without human supervision (also using other technologies such as LTE) [16]. The main idea of this solution is to ensure the connection of all devices in the network and give them a unique number; the so-called UUID (Unified Unique Identifier), which will allow messages to be sent only to a selected node [26].

In order to create a communication tool that will take over the role of a broker, it is necessary to indicate protocols for the exchange of information between two nodes of the platform. The most commonly used protocol in IoT solutions is the MQTT protocol [27]. This solution extends the TCP protocol.

It is also possible to use the WebSocket protocol when implementing the broker [5]. This protocol introduces a full-duplex connection for a TCP connection. It is an extension of the HTTP protocol. The connection is established according to HTTP, where the initiating device sends a request to switch to the WebSocket protocol. During initialization, an additional security key is sent. The server switches the protocol used to WebSocket, maintaining a two-way connection with the client. In the return message, it communicates the redirection and the converted security code. The protocol allows the use of security mechanisms such as authentication or SSL encryption. The described solution has been developed in several projects and is well-known in the literature. Authors present a new approach to involve this concept in novel architecture to be used in the development of a medical data collection system.

**Figure 3:**
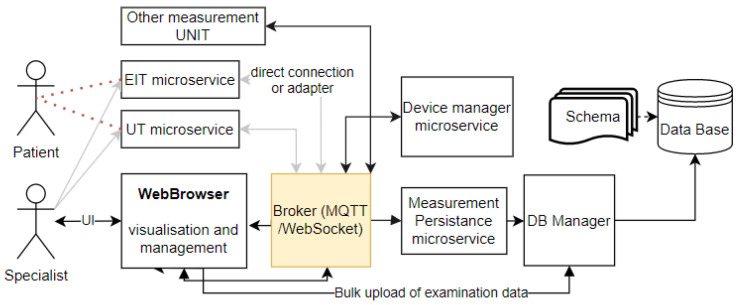
The basic concept of digital platform architecture for medical data collection (context diagram).

Finally, the concept of a system for collecting medical data from tomographic sensors was proposed. It consists of sensors, a message broker, a database manager, a web browser, a database and a database schema. Figure 3 shows the architecture of the solution.

Table 1 presents a proposal of a technology stack as a set of tools necessary for the implementation of a digital platform for collecting medical data.

The most commonly chosen operating systems are Windows and Linux. In server applications, Linux distributions are in the lead, partly due to license and security issues. Due to the use of the Java runtime environment, the indicated aspect is not crucial, and the choice of the system becomes a matter of secondary importance. The solution described in the article is implemented and tested against both solutions.

As noted above, the Java runtime was used. This choice was made due to the fact that this solution introduces the feature of code portability. Equally important was that the language delivered with the platform is strongly typed, which avoids many programming errors at the stage of code compilation. Together with the platform used, frameworks such as Spring and Hibernate were used. The first of them provided dependency inversion mechanisms and mechanisms of database access. The second is responsible for object-relational mapping, with the ability to manage the life cycle of the entity and synchronize access to the database.

The Tomcat application container is a server that allows you to run web applications in Java Servlets, Java Server Pages (JSP) and Java WebSocket technologies. It is one of the most popular open-source web containers. It provides a pure Java HTTP web server environment, supports SSL connections and brings comprehensive authentication mechanism and session management. Tomcat is also a very popular container for standalone applications (not requiring a full application server) written in the Spring Framework. There are also other solutions written in Java, including webservers such as: Wildfly, Jetty, GlassFish, and TomEE, but Tomcat remains sufficient for current needs.

PostgreSQL is an object-relational database management system based on Postgres, developed at the University of California, Berkeley Department of Computer Science. Postgres pioneered many concepts that only became available much later in some commercial database systems. PostgreSQL is an open-source descendant of this original Berkeley code. It supports a large part of the SQL standard and offers many modern features: complex queries, foreign keys, triggers, updatable views, transactional integrity and multi-version concurrency control. As it can be read on a vendors site, due to the liberal license, PostgreSQL can be used, modified, and distributed by anyone free of charge for any purpose, be it private, commercial, or academic. This allows it the opportunity to be used in an innovative solution.

PGAdmin is the most popular and feature-rich open-source administration and development platform for PostgreSQL. The program has a graphical interface and is a good replacement for the psql console program. Currently, from the program level, it is possible, inter alia, to administer databases, database replicas, create any database objects, issue queries and analyze the query plan presented in a classic or graphic form. The choice of administration tool depended on the database that was used. Due to the fact that PGadmin is an application dedicated to work with PostgreSQL, the decision was obvious.

Liquibase is a standalone library for tracking, managing, and applying changes to database schema. The project of this solution started in 2006 to make it easier to track database changes, especially in an agile development environment. This is an open-source solution. The basis of the system’s operation is the ability to store all changes to the data structure. This information is stored in text files, most often XML or SQL, and identified by the filename. Each change in the database structure is marked with the identifier and the name of the author. The list of all applied changes is also stored in the database. Liquibase automatically creates the DatabaseChangeLog Table and DatabaseChangeLogLock Table on first start-up. At subsequent launches, the structure of the database is verified and, if necessary, updated. As a result, there is no database version number, but this approach allows you to work in environments with multiple developers and code branches. The alternative for this tool could be flyway. Despite the fact that both have many features in common, Liquibase provides a much more comprehensive solution to maintain DB structure.

Message brokers are used to transfer messages within the platform. One of them is Mosquitto. As the producer writes on its website: “Eclipse Mosquitto is an open source (EPL/EDL licensed) message broker that implements the MQTT protocol versions 5.0, 3.1.1 and 3.1. Mosquitto is lightweight and is suitable for use on all devices from low power single board computers to full servers.” Numerous sources indicate the significant advantage of the indicated tool: the speed of message transfer. Many studies consider them to be the fastest broker. Mosquitto also has the ability to send messages using the WebSocket protocol. This is ensured by the use of dedicated techniques such as plug-ins.

JavaScript Object Notation (JSON) is a lightweight, text-based, language-independent data interchange format. It was derived from the ECMAScript Programming Language Standard (ECMA-262). JSON defines a small set of formatting rules for the portable representation of structured data. Standard defines the possible writing of an object, which consists of basic elements such as: object type, array, value, string, number and whitespace. Thanks to the use of JSON, it is possible to communicate between the system’s modules using the coding standard that can be read both by the machine and by humans.

The architectural solution of the digital medical data collection platform is based on the MVVC pattern. This approach allows us to separate the application logic layer (business logic) from the presentation layer. In addition, it provides a division between the data structure of internal messages and messages sent and obtained from the data presentation layer. The solution makes the server part modules, which include medical data collection and processing services, independent from the user interface part. In order to implement the software of the presentation layer, it was proposed to use numerous JavaScript libraries. These include jQuery, Angular and Angular material. The pace at which the so-called frontend solutions change forces a flexible approach to code creation, so that you can easily replace the existing code with one that was written in the latest technologies. Currently, technologies based on the reactive programming paradigm are used. This is an extension of the observer pattern and the functional (declarative) programming paradigm. The developed architecture allows us to easily replace the presentation layer using new libraries, such as RxJS.

In 1983, a consortium of ACR (American College of Radiology) and NEMA (National Electrical Manufactures Association) initiated the work on a standard for collecting radiological data, and, in 1985, the first ACR-NEMA 1.0 elaboration was published. In 1993, the standard was released under the name DICOM.

DICOM is a format for recording and exchanging medical image data (Digital Imaging and Communications in Medicine) used all over the world (in Europe-EN standards). A joint DICOM/ISO working group has been established to develop a new standard for Internet access to DICOM objects. The DICOM Standards Committee has a direct link with ISO TC 215. This standard normalizes data transmission between devices of different manufacturers and thus facilitates the use of the obtained image data for several imaging devices and common units.

The DICOM standard facilitates the creation and expansion of PACS (Picture Archiving and Communication Systems) systems, as well as the exchange of information with other IT systems present in medicine (Hospital Information System-HIS). The format of documents saved in this standard is simple and consists of a serially written set of attributes and their values. The structure of a DICOM file consists of a sequence of elements (DICOM PS3.5 2021d-Data Structures and Encoding Page 51). A Data Element is uniquely identified by a Data Element Tag. The Data Elements in a Data Set are ordered by increasing the Data Element Tag Number and occur almost at once in a Data Set. Data types are standard and private, and a Data Element has one of three structures. One of them contains VR field; the other two do not contain such an entry. An example DICOM data element is presented in Table 2.

The development of the standard is continuous, and the work is carried out by 30 teams (Working groups), which include: WG-01 Cardiac and Vascular Information, WG-2 Projection Radiography and Angiography, WG-7 Radiotherapy, WG-12 Ultrasound, WG-14 Security, WG-21 Computer Tomography and WG-23 Application Hosting. The NEMA website http://dicom.nema.org/ (access on 19 October 2021) indicates the specialties in which the described standard applies, including radiology, breast imaging, cardiology, radiotherapy, oncology, ophthalmology, dentistry, pathology, surgery, veterinary, neurology and pneumology. The study described in this article is the basis for extending the list of DICOM applications to the specialty of urological diagnostics using the fusion of Impedance and Ultrasound Tomography. Standard covers documentation consisting of 22 parts. Part 20 describes the integration of DICOM with the new HL7 medical data collection architecture standard.

DWV (DICOM Web Viewer) is an open-source medical security library to visualize DICOM files with zero configuration. It uses technology that uses JavaScript and HTML5, which means you can use the system support that supports your phone, computer, tablet and TV. It can download technical data or zoom in format (standard for technological image data, such as MR, CT, Echo, Mammo, NM) and provides the ability to make tools to manipulate them, such as contrast, zoom, drag, draw, add at the top of the image and image filtering, such as threshold and sharpen [28].

### 2.2. Database Model

The Reference Information Model (RIM) is the cornerstone of the HL7 Version 3 development process. An object model created as part of the Version 3 methodology, the RIM is a large, pictorial representation of the HL7 clinical data (domains) and identifies the life cycle that a message, or groups of related messages, will carry. It is a shared model between all domains and, as such, is the model from which all domains create their messages. The RIM is an ANSI-approved standard [29]. The RIM contains more than 100 entities. It might be dived into several groups that contain organizational data, personal information, service and service event information, some insurance entities and so on. For example, the Person Entity consists of columns such as birth data, birthplace, military_status, religious affiliation, very important person, and preferred_pharmacy. These data are not crucial for our study.

**Figure 4:**
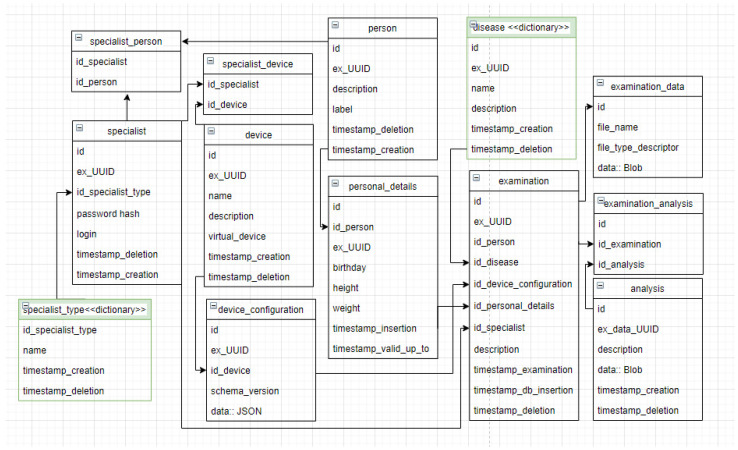
Diagram of a database schema for medical information collecting system.

Instead of using a very extensive model, the authors designed a custom database schema. Figure 4 shows the database structure that was designed and implemented for the collection of a new dataset. A set of tables and relations between them are presented. The diagram describes, inter alia, the tables of data: specialist, measuring device, disease, patient, patient card and examinations. The schema created should be modified in the future to enable integration with systems based on the RIM model to support interoperability. This refers to the necessary data to determine the health of a patient without redundant information.

## 3. Experiments and Results

In order to verify the operation of the prepared digital platform and, particularly, the message broker, a number of efficiency tests were carried out. One of the parameters studied was the transfer time between the platform nodes. The test environment consisted of five deployment entities *A_deployemnt_*. Three web applications (*C*, *P*, *S*), a technical broker *B_c_* and two tested brokers (*B_ws,_ B_mosq_*) are presented in Equations (1)–(3).

(1) $$A_{deployment}=\left\{C,\;P,\;S,\;B_{c},\;B_{1},\;B_{2}\right\}$$

(2) $$B_{1}\in \;B\;\wedge \;B_{2}\;\in \;B$$

(3) $$B=\left\{B_{ws},\;B_{mosq}\right\}$$

The first application *P* (message publisher) simulated a sensor-like UT or EIT. The second one simulated a tool receiving measurement data *S*–subscriber for an instant reconstruction and visualization module. The third one was the application controlling the test process *C* and was responsible for gathering test results and preparing the output from the experiment. During the test, the data transfer time t_PS_(*B_t_*) between two nodes *P* and *S* with use of *B_t_* was measured. This analysis was performed against two factors: the size of transfer message *b* in bytes and number of messages *n* in the experiment (Equation (4)).

(4) $$t_{PS}\left(B_{t},\;n,\;b\right)=\frac{\sum_{x=0}^{n}t{\left(B_{t},b\right)}_{end}-t{\left(B_{t},b\right)}_{start}}{n}$$

**Figure 5:**
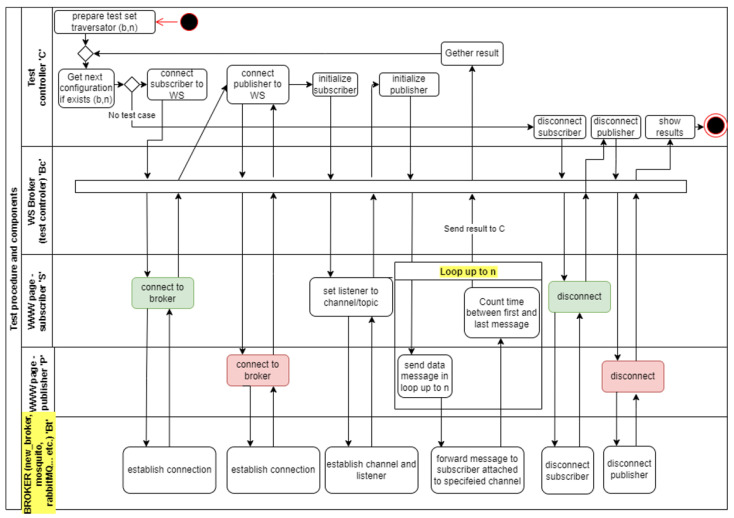
Sequence diagram (UML) that presents broker performance test procedure.

After the test start event was triggered, a sequence of subsequent actions was initiated. The first one involved sending a signal from the *S* application to the tested broker in order to establish a connection. The second event forced the application *P* to bind to the same broker. Then, a signal was sent to subscriber *S*, initiating the process of receiving data from the indicated communication channel. After performing the indicated action, another event started the process of sending data from program *S* to test controller *C*. After finishing the transmission, another signal informed of the completion of the test was sent. This included information about the transmission time *t_PS_* to application *C*. The test sequence diagram is presented in Figure 5.

The runtime environment was the unit consisting of the Intel Core i7-4710HQ processor. The computer was equipped with 32 GB RAM. The platform was installed on the Windows 10 operating system, and the running environment was JRE (Java Runtime Environment) in version: Java 16.0.1.

**Figure 6:**
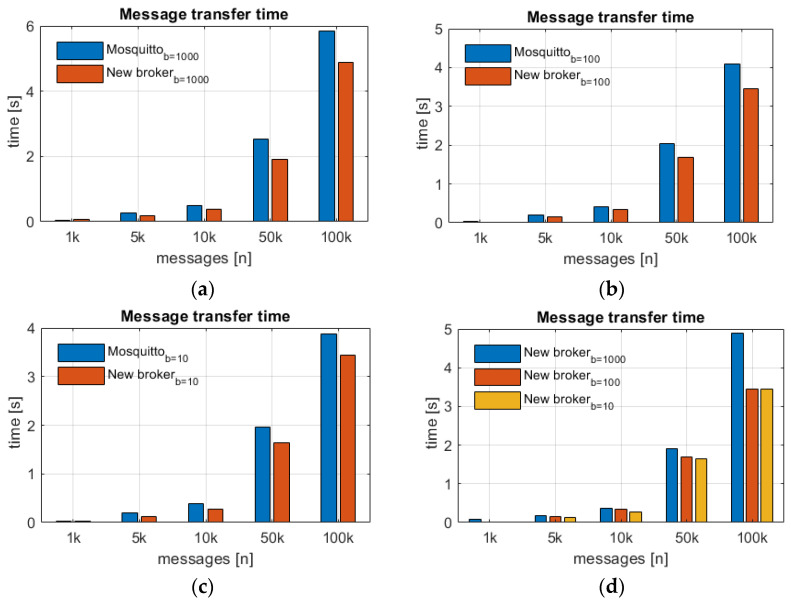
Analysis of brokers’ performance in terms of selected message sizes (parameter b-bytes) and the number of messages sent (*n*); (**a**) message size 1000 bytes; (**b**) message size 100 bytes; (**c**) message size 10 bytes; (**d**) analysis for New Broker.

A series of measurements was carried out for two brokers, which included the “New Broker” and a professional tool used in the industry, i.e., the Mosquitto broker, version 2.0.11. The “New Broker” was prepared in order to handle messages between two nodes in the network. It uses the WebSocket protocol as a core of data exchange. The test procedure was based on sending messages of various sizes (b = 10, 100, 1000 bytes). The time was measured from the receipt of the first message to the receipt of the last message in the stream of prepared data. Tests were performed for sets of 1000, 5000, 10,000, 50,000 and 100,000 messages. The total for each test case combination was repeated 50 times, and the results were averaged. The test results are shown in Figure 6.

The Mosquitto broker was launched in verbose mode, which guaranteed the correctness of data exchange process and the provision of up-to-date information on the current state of the process. The mode unfortunately affected the effectiveness of the tool. Nevertheless, based on the results, the proprietary solution is competitive in relation to the available professional solutions. The above charts indicate that the transfer time for both message brokers is similar and for 100,000 messages it is about 4 s. The characteristic versus the number of messages is linear.

**Figure 7:**
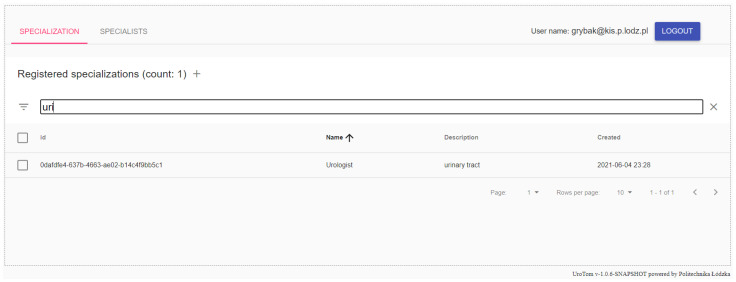
Screenshot—the information management panel about specialists registered in the system (own elaboration).

As part of the work, a digital platform for collecting and processing medical data for tomographic devices was developed. The user interface of the described platform consists of a number of tabs which enable effective management of patient information and measurement data. The tabs correspond to the tables in the diagram presented above, aggregating the measuring device panel, disease panel, patient panel and medical examination panel. The indicated types of data constitute the necessary core that allows the labeling of medical data obtained during a series of studies. The specialist who performs the examination can choose the patient, the disease and the examination method. After indicating these data, it is possible to enter files with test measurement data into the database. The information stored in the database is made available to the internal mechanisms of processing and diagnosing the patient’s health condition. Figure 7 shows a screenshot with an exemplary view of the medical research panel.

**Figure 8:**
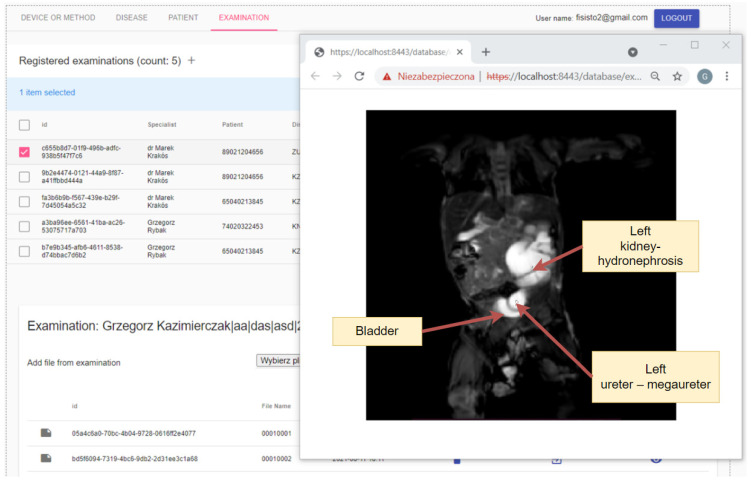
Screenshot of the examination panel with medical image visualization (illustrative image). The graphic shows a screenshot of the window with the demonstration data. The production system has anonymized data.

During the medical examination, it is possible to take measurements using various tools. One may be an ultrasound or impedance tomograph, or it is possible to use other common methods such as magnetic resonance imaging. The prepared system allows the collection of data in the form of DICOM files, the structure of which was described earlier. The graphic below (Figure 8) presents an example of a panel for the visualization of medical data stored in the above-mentioned format. Thanks to the use of web technologies, the user interface has been prepared in such a way as to enable the display of many photos at one time, which in the future will not limit the specialist to using only one application window at a time.

The method that is presented in this article has many benefits, but it also generates some problems and challenges, as mentioned above: services create information barriers, connections between services in the network have a higher cost, testing and implementation are more complicated, transferring functionality between services and system maintenance is more difficult, and the perception of service size as a structuring mechanism may lead to too many microservices. Additionally, the very important case connected with distributed system creation is the security aspect. As the number of nodes grows, the risk of attacks grows and the need to develop mechanisms to protect the system grows.

## 4. Conclusions

The use of digital platforms that allow the collection of medical data, especially from diagnostics, can significantly facilitate the development of new imaging methods based on artificial intelligence. The data obtained from both impedance tomography and ultrasound techniques form the basis of a systems development of a minimally invasive method for the diagnosis of the urinary system. These may be dedicated especially to pediatric patients.

The main attention in this article has been focused on measurements using tomographic sensors and computer infrastructure that allows the process of collected data. The main advantages and disadvantages of the microservices architectural style connected with the IoT concept were indicated. As has been shown, this approach provides many development opportunities and has the advantage of high scalability of the solution. In addition, a new message broker has been prepared that natively supports the WebSocket protocol, which allows devices in the IoT concept to be connected with a web browser through message broker, without the use of additional protocol adapters. Authors provided a description of the technological stack selected for the implementation of the experimental part. Finally, the result of the developed and implemented digital platform for the processing and collection of medical data as applied to tomographic measurements was presented.

In order to improve the prepared system towards expanding functional and quality values, several steps will be taken. For an instance, it is planned to develop mechanisms of the reactive programming paradigm, maintain a compliance with the EHR storage and transfer standards and create algorithms of tomography data fusion, in addition to preparing automatic diagnosis algorithms (artificial intelligence).

## Figures and Tables

**Table 1:** The technological stack of creating a digital platform for collecting tomographic medical data.

Nr.	Stack Element	Name	Version
1	Operating system	Windows/Linux	-
2	Runtime environment	Java JDK	16.0.1
3	IoC, Java Persistence API	Spring	4.2.3.RELEASE
4	Object–relational mapping	Hibernate	4.3.6.Final
5	Application container	Tomcat	9.0
6	Database	PostgreSQL	13
7	Database administration tool	PGAdmin	4.13
8	Databse structure management tool	Liquibase	3.4.2
9	Message broker	NewBroker/Mosquitto	2.0.11
10	Communication data format	JSON	-
11	Frontend libraries (javascript)	jquery	1.1.0
		angular	1.5.8
		angular material	1.1.0
12	Dicom files visualisation (DWV)	dwv.min.js	

**Table 2:** An example DICOM data element.

Tag		VR		Value Length	Value
Group Number (16-bit unsigned integer)	Element Number (16-bit unsigned integer)	VR (2 single byte characters)	Reserved (set to 0000 h)	32-but unsigned integer	Even number of bytes containing the Data Element Value encoded according to the VR
2 bytes	2 bytes	2 bytes	2 bytes	4 bytes	Length specified in ‘value length’

## Data Availability

Not applicable.
